# Assessing Underutilization of Mental Health Resources at a Historically Black College and University (HBCU)

**DOI:** 10.21203/rs.3.rs-3760662/v1

**Published:** 2023-12-19

**Authors:** Ahlam A. Ayyad, Thomas J. Maestri, Savannah Harris, Nina Casanova, Hanan Ibrahim

**Affiliations:** Xavier University of Louisiana; Xavier University of Louisiana; Xavier University of Louisiana; Xavier University of Louisiana; Xavier University of Louisiana

**Keywords:** Mental health, resource utilization, HBCU, College Student, barriers, African American

## Abstract

**Objective:**

In 2018, a survey was conducted with students on a Historically Black College and University (HBCU) campus that showed a significant lack of utilization of both on and off campus mental health resources. The primary outcome of this survey is to evaluate lack of utilization of mental health resources at an HBCU to effectively promote student mental wellness.

**Methods:**

A short electronic survey was administered to students to assess underutilization.

**Results:**

Subjects were predominately African American (60.24%) and female (85.53%). Of the 159 surveys completed, 13 responded they have used on campus mental health resources. Approximately 61.5% (8/13) are satisfied or very satisfied with the services. 29 responded they have used off campus mental health resources. Approximately 41.4% (12/29) are satisfied or very satisfied with the services. 62 (39%) responded that time constraint was a barrier faced in utilizing mental health resources. 60 (38%) responded that they did not feel that mental health resources were currently needed. 40 (25%) responded that they were not aware of mental health resources available. There is a significant association between classification and comfort level continuing to utilize mental health resources on or off campus (p = 0.02).

**Conclusions:**

There are multiple barriers that have attributed to the underutilization of mental health resources. According to the results of this survey, the majority of students lacked time to utilize or denied need for any mental health resources. These results will allow for an opportunity to improve utilization of both on and off campus mental health resources.

## Introduction

According to National Alliance on Mental Illness (NAMI), in 2021 22.8 percent (57.8 million) of United States (US) adults experienced a mental illness; the annual prevalence is 21.4% in African Americans. 47.2 percent of adults with mental illness received treatment, with treatment rates of 39.4 percent in African Americans. Treatment rates in women were reported as 51.4 percent, while male rates were 40 percent.^1^ Results from survey data from the Heatly Minds Study between 2013 and 2021, which included greater than 350,000 students at 373 campuses, reported that greater than 60 percent of students in 2020–2021 met the criteria for one or more mental health problems.^2^ In the US adults aged 18–44, there are nearly 600,000 hospitalizations each year from mood disorders and psychosis spectrum.^1^ Demographics have been linked to the utilization of mental health resources, with older adults being more likely to use resources.^3^

Barriers have been attributed to the lack of utilization of mental health resources. Results from the WHO World Mental Health International College Student Initiative, which assessed barriers in first-year college students, stated that wanting to handle the mental health problem alone, wanting to talk to family or friends instead, or being too embarrassed to seek help were all barriers rated the most important in college students.^4^ In a review conducted by Mahogany S. Anderson, it is stated that even if there are available and accessible resources, African American students are historically less likely to utilize them due to barriers such as stigma, cultural mistrust, or racial/ethnic identity.^3^ African Americans have also utilized coping mechanisms due to the lack of mental health resources available in the community. Another barrier that African Americans face is awareness. Awareness can be the lack of knowledge about mental health resources or the lack of awareness of the location of these resources in the community.^5^ While all of these factors play a part in the overall cause of lack of utilization, each person most likely has their own unique reasoning as to why they do not or will not use the mental health resources that are available to them. The survey conducted through this research aims to examine exactly what that cause is and how students perceive their reasoning for underutilization.

## Methods

A peer-reviewed literature search was done using Google Scholar and PubMed databases between 2001 and 2019. Keywords like “African American” “Mental Health” “Underutilization” and “Barriers” were used to narrow the search. A total of twelve articles were reviewed and determined to be in the scope of this literature review. One hundred fifty-nine surveys were administered to students attending Xavier University of Louisiana to assess mental health resource utilization on campus. The surveys were administered to students throughout the university on a volunteer basis without regard to classification. Each student completed a five-questionnaire survey comprised of questions assessing mental health resources on/off campus, preferred mental health resources, and students’ likeliness to continue utilizing resources.

### Study Design

This was a focused, online survey-based study conducted from September 2022 to November 2022 of students who attended Xavier University of Louisiana. Approval was obtained by Xavier University of Louisiana’s Institutional Review Board. All students who are currently enrolled at Xavier University of Louisiana were eligible for the study. Faculty, staff, and other members of the community were excluded from this study.

### Statistical Analysis

SPSS was used to analyze data in order to assess the utilization of mental health resources amongst students on an HBCU campus.

## Results

159 participants were included in this study. 68 of the students were under 21 years of age, while 91 of the students were 21 years of age or older. 85.53% (136) of the participants were female and 13.84% (22) were male. 1 participant responded as non-binary. 60.24% (100) of the students were Black or African American, while 25.90% (43) were White American, 9.64% (16) were Asian, and 1.20% (2) were American Indian/Alaska Native. 3.01% (5) chose not to disclose race. 86.54% (135) of the students were also non-Hispanic or Latino, with 9.64% (15) being Hispanic or Latino. 3.85% (6) chose not to disclose ethnicity. The classification of participants within the study was spread throughout each level, with it being split between 87 graduate students and 72 undergraduate students and having a p-value of less than 0.05.

Participants had the option to describe the barriers they faced to utilization of mental health resources through a multiple-choice style question, as well as an “other” box to type their response. The following answers resulted from this question. 39 students chose not to answer. 12 students answered that there was concern regarding judgement if resources were utilized. 62 students answered that they did not feel resources were needed at the time. 40 students answered that they were not aware of current available resources. 64 students answered that they felt a time constraint. 5 students specified other reasons. Other reasons included that they convinced themselves the resources were for people with bigger problems, there was a language barrier, they have tried to use resources before and they did not help, as well as there being a lack of money and they did not feel like sitting down to talk about their feelings.

Another question in the survey asked participants what forms of coping mechanisms they found most beneficial to them. 115 participants prefer to speak to friends and/or family members. 65 participants prefer to handle the situation on their own. 32 prefer to utilize mental health resources. 13 participants prefer not to disclose. 3 participants specified other methods, including anxiety medication, as well as meditation, journaling, and art.

Lastly, participants were asked to rate their level of comfort in seeking help or continuing to utilize mental health resources on or off campus on a scale of 1–10. 16 participants responded they were not at all comfortable, whereas 26 participants responded that they were completely comfortable.

## Discussion

Students were predominately African American (60.24%) and female (85.53%). These statistics are consistent with the demographics of the University at study being mostly African American female students. Of the 159 surveys completed, 13 responded they have used mental health resources on campus. Approximately 61.5% (8/13) are satisfied or very satisfied with the services. 29 responded they have used off campus mental health resources. Approximately 41.4% (12/29) are satisfied or very satisfied with the services. 62 (39%) responded that time constraint was a barrier faced in utilizing mental health resources. 60 (38%) responded that they did not feel that mental health resources were currently needed. 40 (25%) responded that they were not aware of the mental health resources available. There is a significant association between classification and comfort level continuing to utilize mental health resources on or off campus (p = 0.02).

### Limitations

Limitations include a lack of a validated tool to assess utilization. Additionally, this data may not be fully representative of the population on campus. As some students may not feel comfortable in busier parts of campus, and those interested in mental health may have been more willing than others to participate in the survey. Data collected from a single institution could be reflective of factors pertaining to the institution rather than of HBCUs as a whole. Despite these limitations, this data does reflect similar rates of utilization of African American college students in other settings according to previous literature. This study is still able to illustrate the importance of future focus on mental health utilization in African American students in the HBCU setting.

## Conclusions

This study shows that there are multiple barriers that have been attributed to the underutilization of mental health resources both on and off campus. According to the results of this survey, the majority of students lack time to utilize or denied the need for any mental health resources. Further research should be conducted regarding the association between classification and comfort level. Further research could also be done to gather the best way to promote the various mental health resources that are available that could benefit different individuals. These results will allow for an opportunity to improve utilization of both on and off-campus mental health resources.

## Figures and Tables

**Figure 1:**
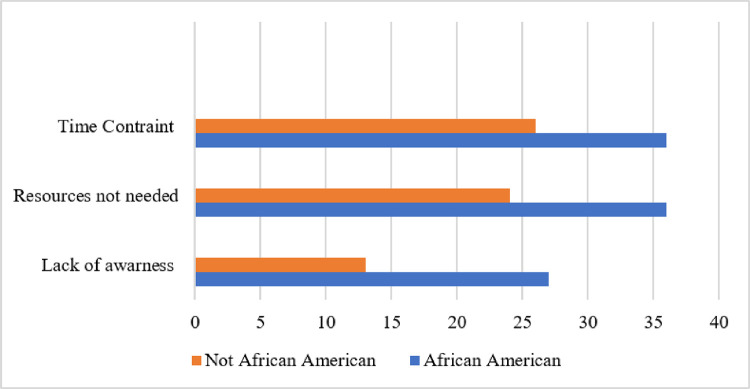
Most Frequent Barriers to Underutilization

**Table 1:** Baseline Characteristics

Baseline Characteristics Survey (n = 159)	

**Age**	**< 21: 68** **≥ 21:91**

**Gender**	Female: 85.53% (136) Male: 13.84% (22) Non-binary: 0.63% (1) Transgender: 0.00% (0) Choose not to disclose: 0.00% (0)

**Race**	Black/African American: 60.24% (100) American: 25.90% (43) White: 25.90% (43) Asian: 9.64% (16) American Indian/Alaska Native: 1.20% (2) Choose not to disclose: 3.01 % (5)

**Ethnicity**	Hispanic/Latino: 9.62% (15) Non-Hispanic/Latino: 86.54% (135) Choose not to disclose: 3.85% (6)

**Classification**	Graduate: 87 Undergraduate: 72

**Table 2:** Utilization

Utilization					
Category	Sub-Category	On-Campus	P-Value Odds Ratio	Off-Campus	P-Value Odds Ratio
**Age**	< 21	4	P = 0.362 OR = 0.57 with 95% CI 0.2–2	12	P = 0.867 OR = 0.93 with 95% CI 0.4–2
	≥ 21	9		17
**Gender**	Female	12	P = 0.275 OR = 2.0 with 95% CI 0.5 to 8	26	P = 0.205 OR = 2.4 with 95% CI 0.5–10.8
	Male	1		2
**Race**	African American	10	P = 0.275 OR = 2.0 with 95% CI 0.5 to 8	19	P = 0.746 OR = 1.15 with 95% CI 0.5–2.7
	Non-African American	3		10	
**Ethnicity**	Hispanic/Latino	1	P = 0.647 OR = 0.8 with 95% CI 0.1 –6.5	4	P = 0.281 OR = 1.7 with 95% CI 0.5–6
	Non-Hispanic/Latino	12		25	
**Classification**	Graduate	8	P = 0.606 OR = 1.3 with 95% CI 0.5–4	16	P = 0.957 OR = 1.02 with 95% CI 0.46–2.3
	Undergraduate	5		13	

## References

[1] Mental Health by the Numbers - NAMI, nami.org/NAMI/media/NAMI-Media/Infographics/NAMI_2020MH_ByTheNumbers_Adults-r.pdf. Accessed 30 Apr.2023.

[2] Lipson S. K., Zhou S., Abelson S., Heinze J., Jirsa M., Morigney J., Patterson A., Singh M., & Eisenberg D. (2022). Trends in college student mental health and help-seeking by Race/ethnicity: Findings from the National Healthy Minds Study, 2013–2021. Journal of Affective Disorders, 306, 138–147. 10.1016/j.jad.2022.03.038. 35307411 PMC8995361

[3] Anderson M. S. (2018). Barriers to the Utilization of Mental Health Services on College Campuses by African-American Students. McNair Scholars Research Journal: Vol. 11, Article 3.

[4] Ebert D. D., Mortier P., Kaehlke F., Bruffaerts R., Baumeister H., Auerbach R. P., Alonso J., Vilagut G., Martínez K. U., Lochner C., Cuijpers P., Kuechler A., Green J., Hasking P., Lapsley C., Sampson N. A., & Kessler R. C. (2019). Barriers of mental health treatment utilization among first-year college students: First cross-national results from The Who World Mental Health International College Student initiative. International Journal of Methods in Psychiatric Research, 28(2). 10.1002/mpr.1782. PMC6522323 31069905

[5] Wilson S. L., Gaunt C. J., Jones K. N., & Solomon C. (2018). Correlation Between Perception and the Underutilization of Mental Health Services in the Treatment of Depression Amongst African Americans. EC Psychology and Psychiatry, 7(5), 263–269.

[6] Eisenberg D., Hunt J., & Speer N. (2012). Help seeking for mental health on college campuses: Review of Evidence and next steps for research and Practice. Harvard Review of Psychiatry, 20(4), 222–232. 10.3109/10673229.2012.712839. 22894731

[7] Nash S., Sixbey M., An S., & Puig A. (2017). University students’ perceived need for mental health services: A study of variables related to not seeking help. Psychological Services, 14(4), 502–512. 10.1037/ser0000172. 29120208

[8] Czyz E. K., Horwitz A. G., Eisenberg D., Kramer A., & King C. A. (2013). Self-reported barriers to professional help seeking among college students at elevated risk for suicide. Journal of American College Health, 61(7), 398–406. 10.1080/07448481.2013.820731. 24010494 PMC3788673

[9] Lannin D. G., Vogel D. L., Brenner R. E., Abraham W. T., & Heath P. J. (2016). Does self-stigma reduce the probability of seeking mental health information? Journal of Counseling Psychology, 63(3), 351–358. 10.1037/cou0000108. 26323042

[10] Masuda A., Anderson P. L., & Edmonds J. (2012). Help-Seeking Attitudes, Mental Health Stigma, and Self-Concealment Among African American College Students. Journal of Black Studies, 43(7), 773–786. 10.1177/0021934712445806.

